# Intersection of hippocampus and spinal cord: a focus on the hippocampal alpha-synuclein accumulation, dopaminergic receptors, neurogenesis, and cognitive function following spinal cord injury in male rats

**DOI:** 10.1186/s12868-022-00729-5

**Published:** 2022-07-12

**Authors:** Ahad Karimzadeh Kalkhoran, Mohammad Reza Alipour, Mohsen Jafarzadehgharehziaaddin, Hamid Soltani Zangbar, Parviz Shahabi

**Affiliations:** 1 Drug Applied Research Center, Tabriz University of Medical Sciences, Golgasht Street, Tabriz, 51666-14766 East Azarbayjan Iran; 2 Department of Neuroscience and Cognition, Faculty of Advanced Medical Sciences, Tabriz University of Medical Sciences, Golgasht Street, Tabriz, East Azarbayjan Iran; 3 Department of Education and Psychology, University of Aveiro, Campus Universitario de Santiago, Aveiro, Portugal

**Keywords:** Spinal cord injury, Hippocampus, Neurogenesis, Alpha-synuclein, Cognition, Dopaminergic receptors

## Abstract

**Background:**

Following Spinal Cord Injury (SCI), innumerable inflammatory and degenerative fluctuations appear in the injured site, and even remotely in manifold areas of the brain. Howbeit, inflammatory, degenerative, and oscillatory changes of motor cortices have been demonstrated to be due to SCI, according to recent studies confirming the involvement of cognitive areas of the brain, such as hippocampus and prefrontal cortex. Therefore, addressing SCI induced cognitive complications via different sights can be contributory in the treatment approaches.

**Results:**

Herein, we used 16 male Wistar rats (Sham = 8, SCI = 8). Immunohistochemical results revealed that spinal cord contusion significantly increases the accumulation of alpha-synuclein and decreases the expression of Doublecortin (DCX) in the hippocampal regions like Cornu Ammonis1 (CA1) and Dentate Gyrus (DG). Theses degenerative manifestations were parallel with a low expression of Achaete-Scute Family BHLH Transcription Factor 1 (ASCL1), SRY (sex determining region Y)-box 2 (SOX2), and dopaminergic receptors (D1 and D5). Additionally, based on the TUNEL assay analysis, SCI significantly increased the number of apoptotic cells in the CA1 and DG regions. Cognitive function of the animals was assessed, using the O-X maze and Novel Object Recognition (NORT); the obtained findings indicted that after SCI, hippocampal neurodegeneration significantly coincides with the impairment of learning, memory and recognition capability of the injured animals.

**Conclusions:**

Based on the obtained findings, herein SCI reduces neurogenesis, decreases the expression of D1 and D5, and increases apoptosis in the hippocampus, which are all associated with cognitive function deficits.

**Graphical Abstract:**

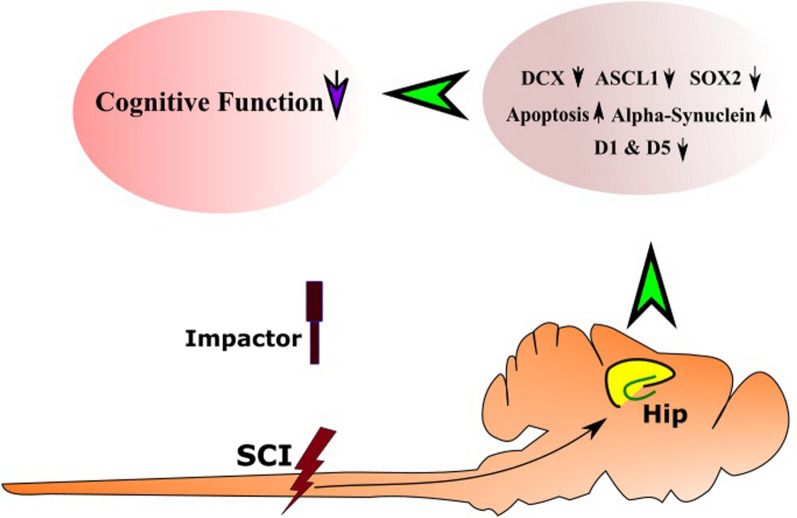

## Background

Numerous individuals complain about cognitive decline following Spinal Cord Injury (SCI), which significantly affect the quality of their life [1]. Physical, depressive-like and psychological complications of SCI have been broadly studied in animal models [2]. This evidence of post-injury cognitive dysfunctions in experimental models of SCI has indicated that physiological alterations inherent to SCI may lead to a rise in cognitive deficits.

A number of studies have shown that following SCI, proinflammatory cytokines like Interleukin-1 (IL-1) and Tumor Necrosis Factor-α (TNF-α) are elevated in the injured spinal cord [3]. Moreover, the brain of injured animals is not immune to these inflammatory changes accordingly, SCI impresses cell cycle function and motives widespread microglial activity in diverse brain areas, with increased expression of cell-cycle-related proteins (Cyclin Dependent Kinase4 (CDK4) and cyclin D1) and genes (cyclins A1, A2, E2F1, D1, and PCNA). On the other hand, these inflammatory changes accompany with recognition deficiency that evaluated with NORT, and spatial memory deficiency that evaluated with Y-Maze and Morris water maze (MWM) [4]. An important point is that, after the SCI, the glial and inflammatory reactivity are generally prominent in cognition-related parts of the brain especially hippocampus [5]. On the flip side, there is a close interplay between the neuroinflammatory factors and aggregation of α-synuclein through progression of cognitive disorders like Alzheimer’s and Parkinson’s disease [6, 7], and this accumulation occurs in parallel with the dysfunction of dopaminergic system [8]. Dopaminergic receptors (D1, D2, D3, D4, D5) are vital in the hippocampus-dependent cognitive functions, such that, modulation of D1 receptors in the hippocampal circuits are linked to the integration of spatial memory and executive functions [9]. Furthermore, pharmacological findings have confirmed that D_1_-like receptors (D_1_ and D_5_) are critically involved in cognitive functions, and D_5_ mutant mice have a deficit in the regulation of spatial working memory and temporal order of memory [10].

In addition to inflammatory changes, apoptotic changes have been seen in the brain following SCI, particullary in the sensory motor cortex [11, 12]. Despite motor areas, SCI produces a significant apoptosis in the hippocampal regions, like Cornu Ammonis1 (CA1) and CA3 [13]. Alongside the inflammatory and apoptotic changes, Dentate Gyrus (DG) as a neurogenic part of the hippocampus is affected after SCI [14]. DG of the hippocampus is an area where sensory information merge together to form a unique representation of these modalities; thus, it plays a pivotal role in learning and memory [15, 16]. The sub-granular zone (SGZ) of DG is an important area that preserves its neurogenic nature from the embryonic period to adulthood [17]. Neural stem cells of SGZ, mature under the influence of various factors, such as Achaete-Scute Family BHLH Transcription Factor 1 (ASCL1) and Doublecortin (DCX), until they eventually home in the DG, in the form of granular cells [18–20]. Following SCI, besides cell loss in different areas of the hippocampus, the expression of DCX^+^ cells decrease in the hippocampus, confirming the attenuation of hippocampal neurogenesis due to SCI [21]. All these destructive changes can be accompanied by deficiency in various aspects of cognitive function such as spatial memory and recognition capability [22].

In the present study, we specifically suggested that α-synuclein aggregation and deficiency of hippocampal dopaminergic system in parallel with neurodegeneration and apoptosis may underlie the development of cognitive dysfunctions, after SCI.

## Results

### Mild SCI impairs motor function but the locomotion improves over the weeks

**Fig. 1:**
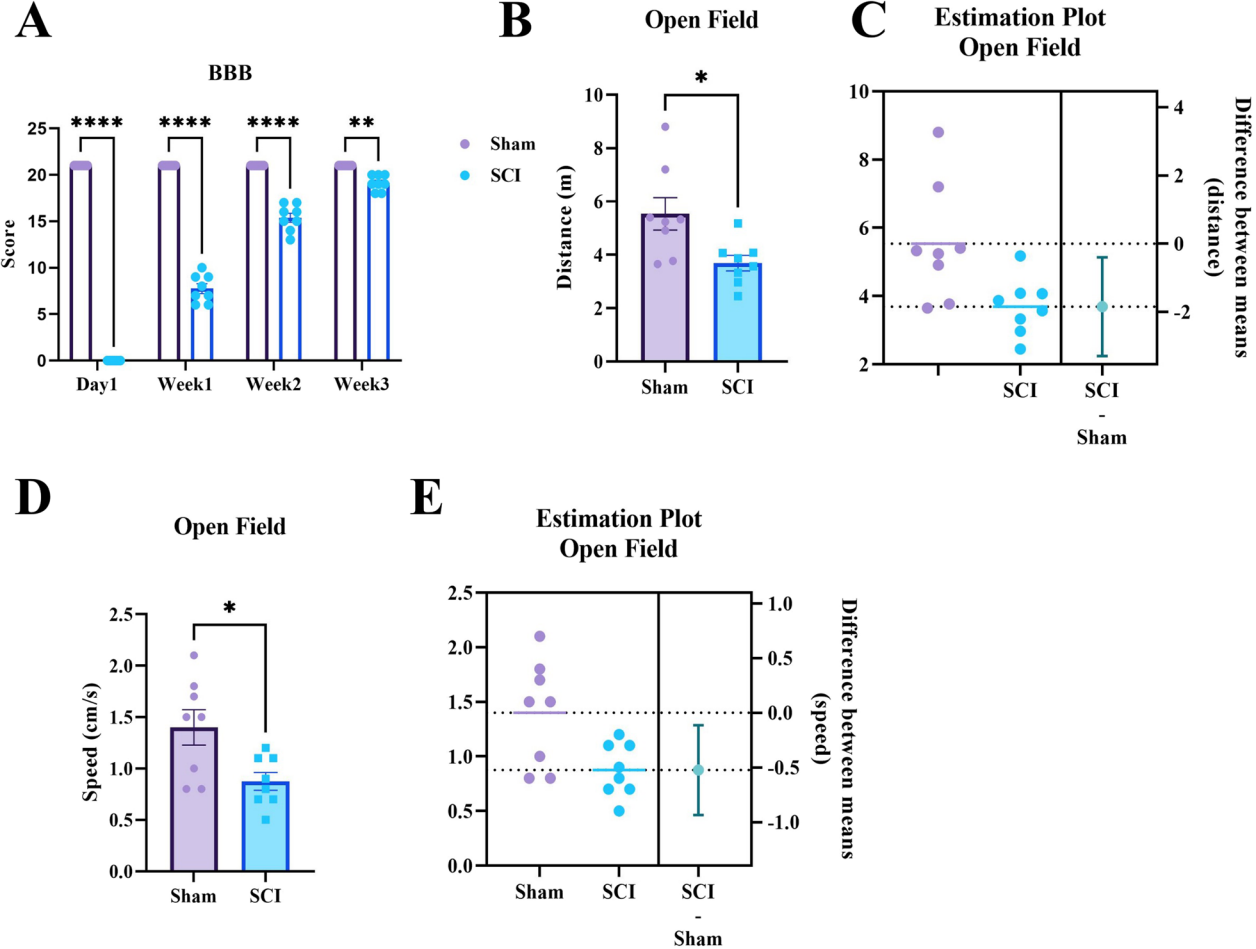
Mild SCI impairs motor function but the locomotion improves over the weeks. **A** The motor function of the hind limbs was assessed using the BBB scale on the first day, first week and second week. Despite the improved function of the injured animals, their hind-limbs motor function was significantly weaker than the sham animals in all three stages of evaluation (**P < 0.01, ***P < 0.001, ****P < 0.0001). **B** The traveled distance by animals, through gait, was assessed using the Open field test. Injured animals had a significantly lower traveled distance in contrast to sham animals (*P < 0.05). **C** Estimation plot of traveled distance with 95% Confidence Interval (CI). **D** The speed of animals’ locomotion through the gait was assessed using the open field test, also. As with traveled distance, the speed of locomotion was significantly lower in injured animals (*P < 0.05). **E** Estimation plot of speed with 95% Confidence Interval (CI)

The hind limbs function of all rats was assessed during the gait, for three consecutive weeks, using the BBB scale. Two-way repeated measures ANOVA, showed a significant effect of weeks (F (2.329, 32.60) = 738.5, P < 0.0001), and groups (F (1, 14) = 1365, P < 0.0001). Injured animals had a poor locomotion on the first day (P < 0.0001), first week (P < 0.0001), second week (P = 0.0001), and third week (P = 0.0015) compared to sham animals (Fig. 1A). However, at the end of the third week, the motor function of the injured animals had improved and they were able to participate in cognitive tests. Also, according to the open field test, at the end of the third week, despite the significant difference between the groups in term of traveled distance (P = 0.0158, t (14) = 2.744) (Fig. 1B and C), and speed of locomotion (P = 0.0159, t (14) = 2.741) (Fig. 1D and E), they were able to participate in cognitive tests.

### SCI impairs cognitive function

**Fig. 2:**
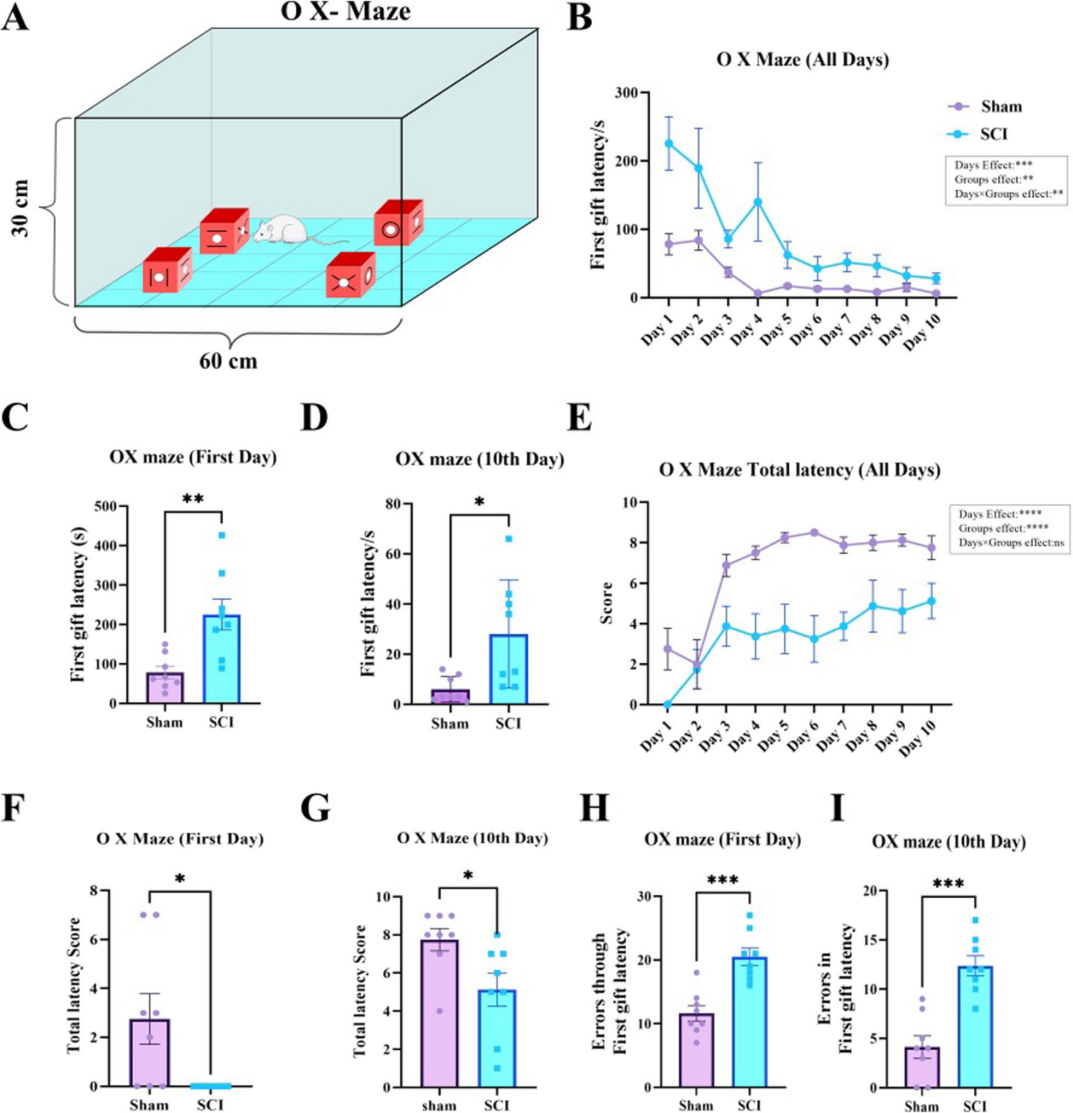
SCI impairs learning and memory which was determined by O-X maze test. **A** O-X maze apparatus. **B** Two-way repeated measures ANOVA of the first gift latency, showed that there is a significant effect of days, groups, and days × groups (**P < 0.01, ***P < 0.001). **C** and **D** The performance of injured animals in the first gift latency was significantly weaker compared to sham animals in both the first and last days (*P < 0.05, **P < 0.01). **E** The Two-way repeated measures ANOVA of total latency, showed that there is a significant effect of days, groups, and no significant effect of days × groups (****P < 0.0001). **F** and **G** The performance of injured animals in the total latency was significantly weaker compared to sham animals in both the first and last days (*P < 0.05). **H** and **I** The number of errors, through first gift latency, in the SCI group was significantly higher in SCI group compared to control group, in both first and last days (***P < 0.001)

The NORT and O-X maze were used to evaluate the effect of SCI on cognitive function of animals. At the end of the third week, following the improvement of injured rats’ locomotion, the animals’ memory and learning were assessed using the O-X maze test. Based on the two-way repeated measures ANOVA of the first gift latency, there was a significant effect of days (F (1.673, 23.42) = 12.84, P = 0.0003), groups (F (1, 14) = 10.37, P = 0.0062), and days × groups (F (9, 126) = 3.146, P = 0.0019). However, over time, the animals found the first reward faster, but on the both first (P = 0.0035, t (14) = 3.503), and last day (tenth) (P = 0.0137, t (14) = 2.817), the function of injured animals was significantly weaker than control animals, so that they spent more time to find the first gift (Fig. 2B–D). Also, according to the two-way repeated measures ANOVA of total latency, there was a significant effect of days (F (3.609, 50.52) = 12.32, P < 0.0001), groups (F (1, 14) = 31.22), and no significant effect of days × groups (F (9, 126) = 1.617, P = 0.1172). As the days went by, the animals of both groups completed the task faster, but as in the first gift latency, the injured animals performed worse than the control animals on both the first (P = 0.0184, t (14) = 2.668) and last (P = 0.0261, t (14) = 2.487) days (Fig. 2E–G). Also, the number of errors, through first gift latency, in the SCI group was significantly higher than the control group, both on the first day (P = 0.0002, t (14) = 4.871) and last day (P = 0.0001, t (14) = 5.358) (Fig. 2H, I).

**Fig. 3:**
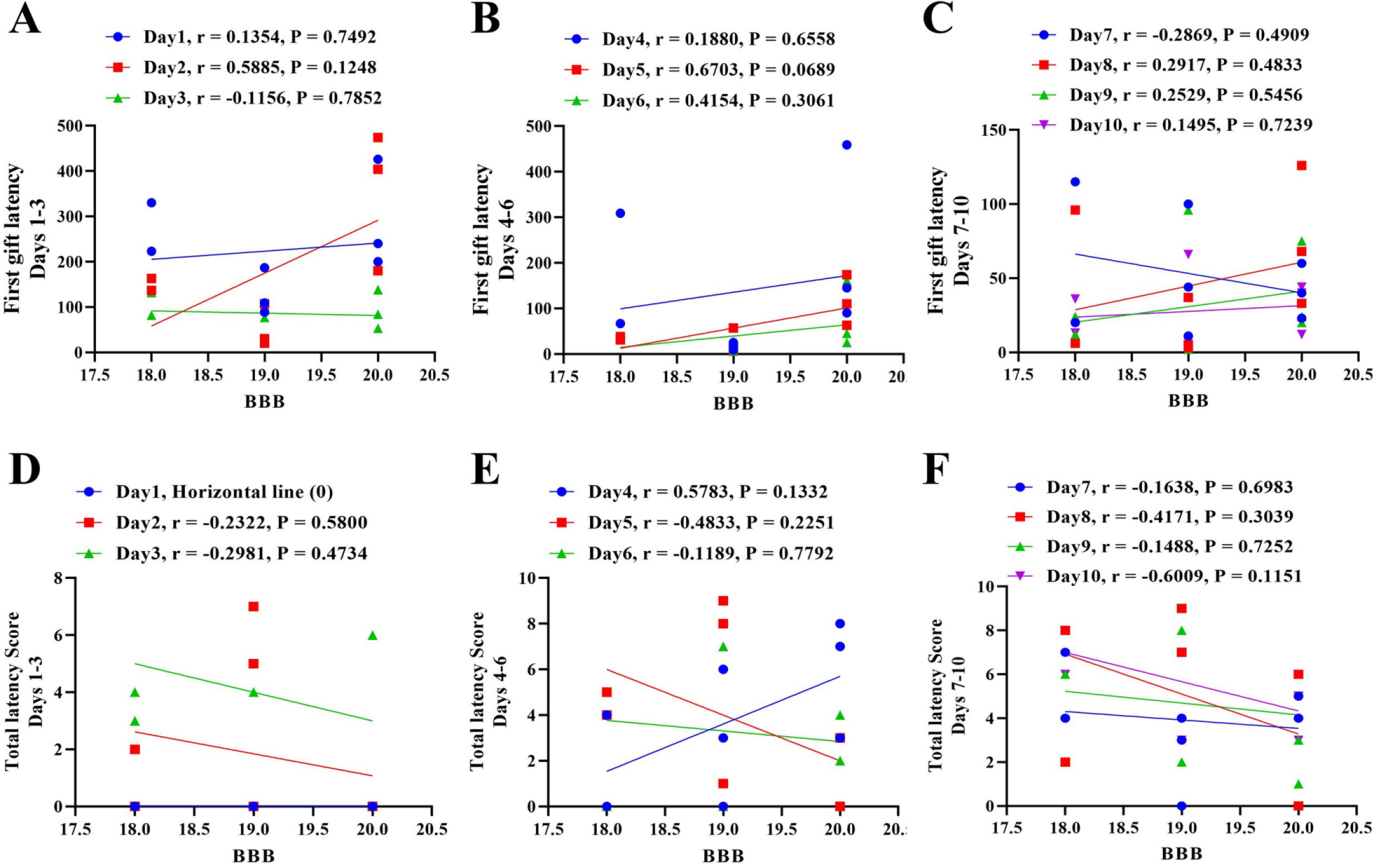
There is no predictive correlation between the hind limb locomotion of injured animals and O-X maze scores. **A**–**C** Linear regression showed there is no significant predictive association between the first gift latency of O-X maze and BBB scores of injured animals on any day of the test. **D**–**F** Linear regression showed there is no significant predictive association between the total latency scores of O-X maze and BBB scores of injured animals on any day of the test

Considering that the deficiency of hind limb locomotion could be an interfering factor in the cognitive function of injured group, so linear regression was used to evaluate the predictive effect of BBB scores (last day recovery scores) on O-X maze indices (First gift latency and Total latency), in SCI group. Regression results showed that in none of the first to tenth days of O-X maze test, there was no significant predictive association between the O-X maze indices and BBB scores (Fig. 3A–F). Considering BBB score as a covariate, we analyzed the significancy of its adjustment effect on animals’ cognitive function in the O-X maze, using ANCOVA. The results showed that the locomotion of animals in the first, second, third and fifth days, had significant effect on their performance in the first gift latency (Table 1). Also, the locomotion of animals significantly affected the total latency score of animals in the third, fourth, sixth and seventh days (Table 2).

**Table 1:** BBB score as a covariate in the assessment of first gift latency

O-X Maze	BBB adjusted effect	
	F	P value
First gift latency—Day 1	5.8	0.01
First gift latency—Day 2	5.2	0.02
First gift latency—Day 3	9.5	0.003
First gift latency—Day 4	2.8	0.09
First gift latency—Day 5	4.7	0.02
First gift latency—Day 6	3.3	0.07
First gift latency—Day 7	3.4	0.07
First gift latency—Day 8	1.1	0.3
First gift latency—Day 9	2.8	0.09
First gift latency—Day 10	3.4	0.1

BBB score covaried (ANCOVA)

**Table 2:** BBB score as a covariate in the assessment of total latency

O-X Maze	BBB adjusted effect	
	F	P value
Total latency—Day 1	3.3	0.07
Total latency—Day 2	0.15	0.8
Total latency—Day 3	9.7	0.003
Total latency—Day 4	8.9	0.02
Total latency—Day 5	4.5	0.06
Total latency—Day 6	12	0.001
Total latency—Day 7	5.3	0.02
Total latency—Day 8	6.4	0.08
Total latency—Day 9	3.9	0.05
Total latency—Day 10	7.8	0.09

BBB score covaried (ANCOVA)

**Fig. 4:**
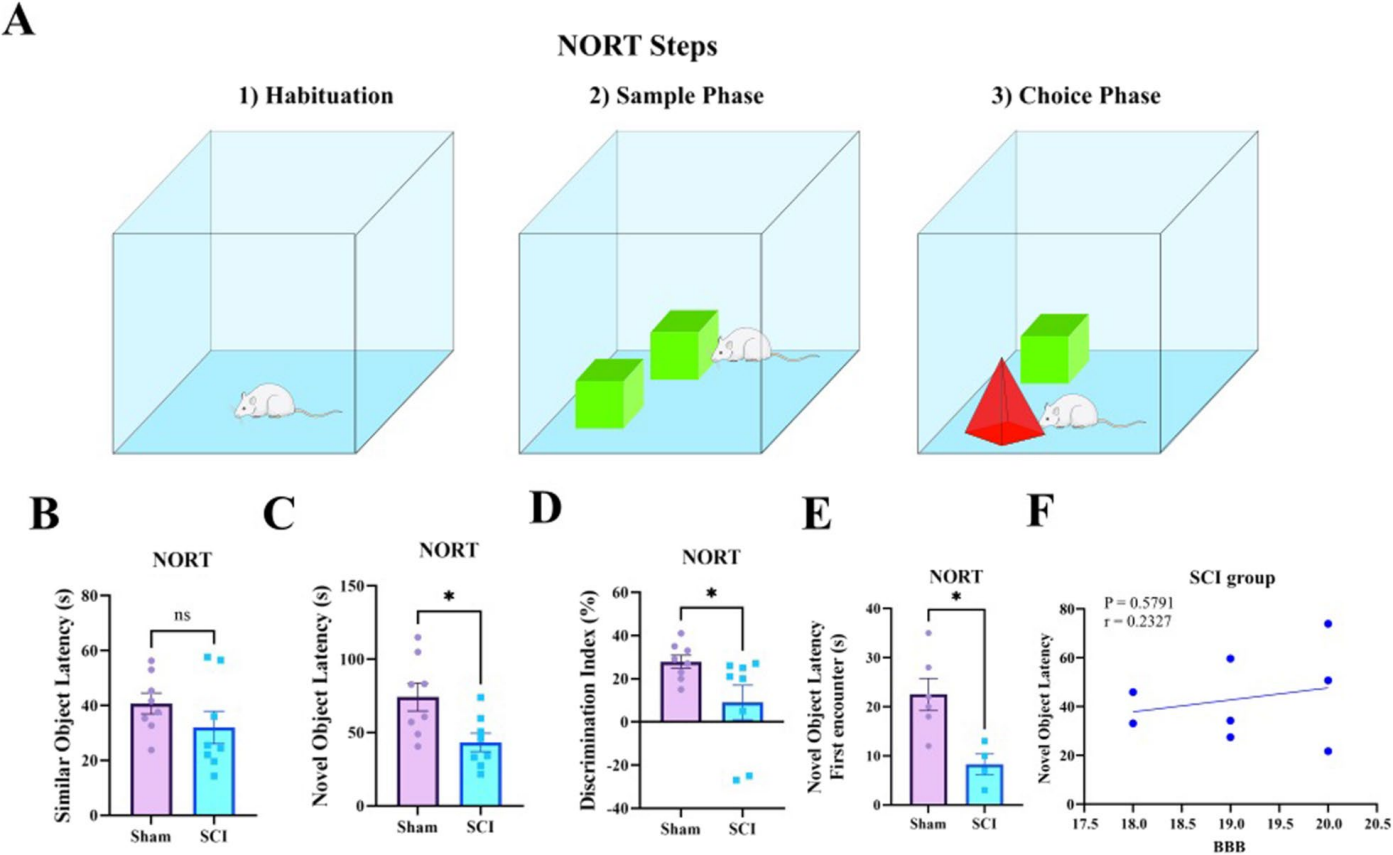
SCI impairs recognition which was determined by NORT. **A** Phases of NORT. **B**–**D** There was no significant difference between groups in the similar object latency through choice phase, but the novel object latency and discrimination index were significantly difference between them (*P < 0.05, ns: not significant). **E** The animals of SCI group spent significantly less time with novel object compared to animals of control group through choosing the novel object as a first choice in the choice phase of the NORT (*P < 0.05). **F** Linear regression showed there is no significant predictive association between the novel object latency and BBB

After the O-X maze test, the animals rested for three days and the recognition capability of the animals was assessed using NORT test, from day 35 to 37. The results of independent t-test indicate that through the choice phase, the novel object latency of SCI group was significantly lower than control group (P = 0.0163, t (14) = 2.728) (Fig. 4C), however there was no significant difference in the similar object latency (P = 0.2340, t (14) = 1.244) (Fig. 4B). In general, considering the discrimination index, the recognitive ability of rats significantly reduced after the SCI (P = 0.0447, t (14) = 2.205) (Fig. 4D). Also, among the animals in the SCI group, 4 animals and among the animals in the control group, 6 animals moved toward the novel object in the first choice of the choice phase. The comparison of the novel object latency, as a first choice, showed that the animals in the SCI group spent significantly less time with novel object than the animals in the control group (P = 0.0124, t (8) = 3.211) (Fig. 4E). Like O-X maze, linear regression was used to evaluate the predictive effect of BBB scores (last day recovery scores) on novel object latency, in SCI group. Regression results showed that, there was no significant predictive association between the novel object latency and BBB scores (Fig. 4F). Considering BBB score as a covariate in the ANCOVA, it had no significant effect on the novel object latency and discrimination index (Table 3).

**Table 3:** BBB score as a covariate in the assessment of novel object latency and Discrimination index

NORT	BBB adjusted effect	
	F	P value
Novel object latency	3.6	0.06
Discrimination index	2.6	0.1

BBB score covaried (ANCOVA)

### SCI decreases the expression of D1 receptor, D5 receptor, SOX2 and ASCL1

**Fig. 5:**
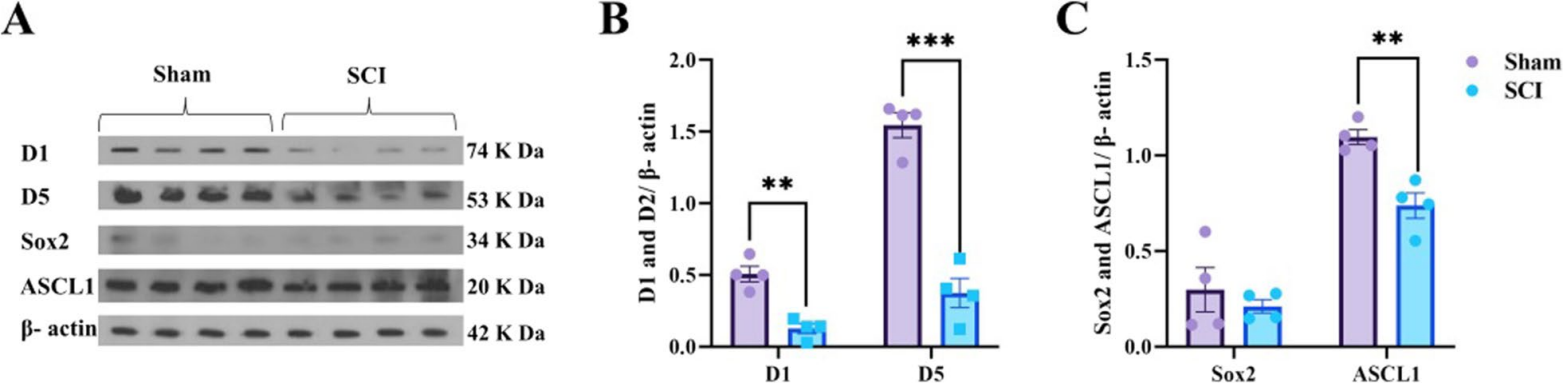
SCI decreases the expression of D1 receptor, D5 receptor, SOX2 and ASCL1. **A** Immunoblotting cropped images of D1 receptor, D5 receptor, SOX2 and ASCL1. **B** Quantitative densitometric analysis of D1 and D5 receptors (n = 4 per group, **P < 0.01, ***P < 0.001, ****P < 0.0001). **C** Quantitative densitometric analysis of SOX2 and ASCL1 (n = 4 per group, *P < 0.05, **P < 0.01)

The expression level of D1 receptor, D5 receptor, SRY (sex determining region Y)-box 2 (SOX2) and ASCL1, which are important in the cognitive function and neurogenesis of hippocampus, were assessed by western blotting in both sham (n = 4) and SCI groups (n = 4) (Fig. 5A). According to the independent t-test, the expression level of D1 receptor (P = 0.0010, t (6) = 5.918) and D5 receptor (P = 0.0001, t (6) = 8.750) was statically significant between groups (Fig. 5B). Also, there was a significant difference between groups in term of ASCL1 expression (P = 0.0035, t (6) = 4.655), however, despite the reduced expression of SOX2 in the SCI group, there was no significant difference (P = 0.4980, t (6) = 0.7210) in the expression level of that between groups (Fig. 5C).

### SCI decreases the expression of DCX and increases the aggregation of α-synuclein in the hippocampus

**Fig. 6:**
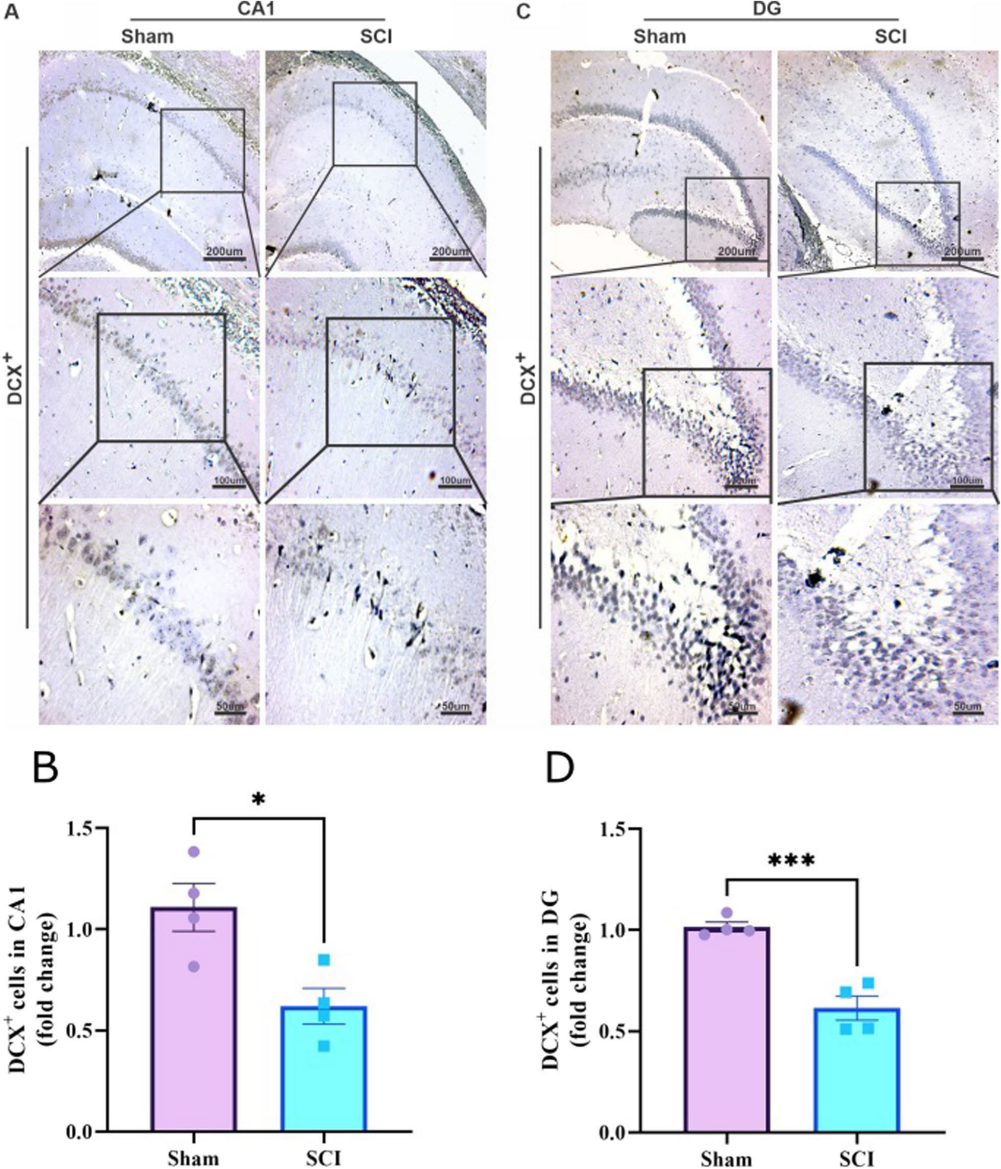
SCI decreases the expression of DCX in the CA1 and DG areas of the hippocampus. **A** Representative immunohistochemical images of DCX^+^ cells in the CA1 of hippocampus. **B** Bar diagram shows that SCI significantly decreased the number of DCX^+^ cells in the CA1 (n = 4 per group, *P < 0.05). **C** Representative immunohistochemical images of DCX^+^ cells in the DG of hippocampus. **D** Bar diagram shows that SCI significantly decreased the number of DCX^+^ cells in the DG (n = 4 per group, ***P < 0.001)

To study the possible distant effect of SCI on the hippocampal neurogenesis and aggregation of α-synuclein in the hippocampal areas, the expression level of DCX and aggregation of α-synuclein were evaluated in the hippocampus, immunohistochemically. Figure 6A and C, are immunohistochemical representations of DCX in the CA1 and DG areas of the hippocampus. In accordance with the independent t-test, there is a significant difference between the groups (n = 4 in both groups) in terms of DCX expression in both CA1 area (P = 0.0164, t (6) = 3.300) and DG area (P = 0.0008, t (6) = 6.267) (Fig. 6B and D).

**Fig. 7:**
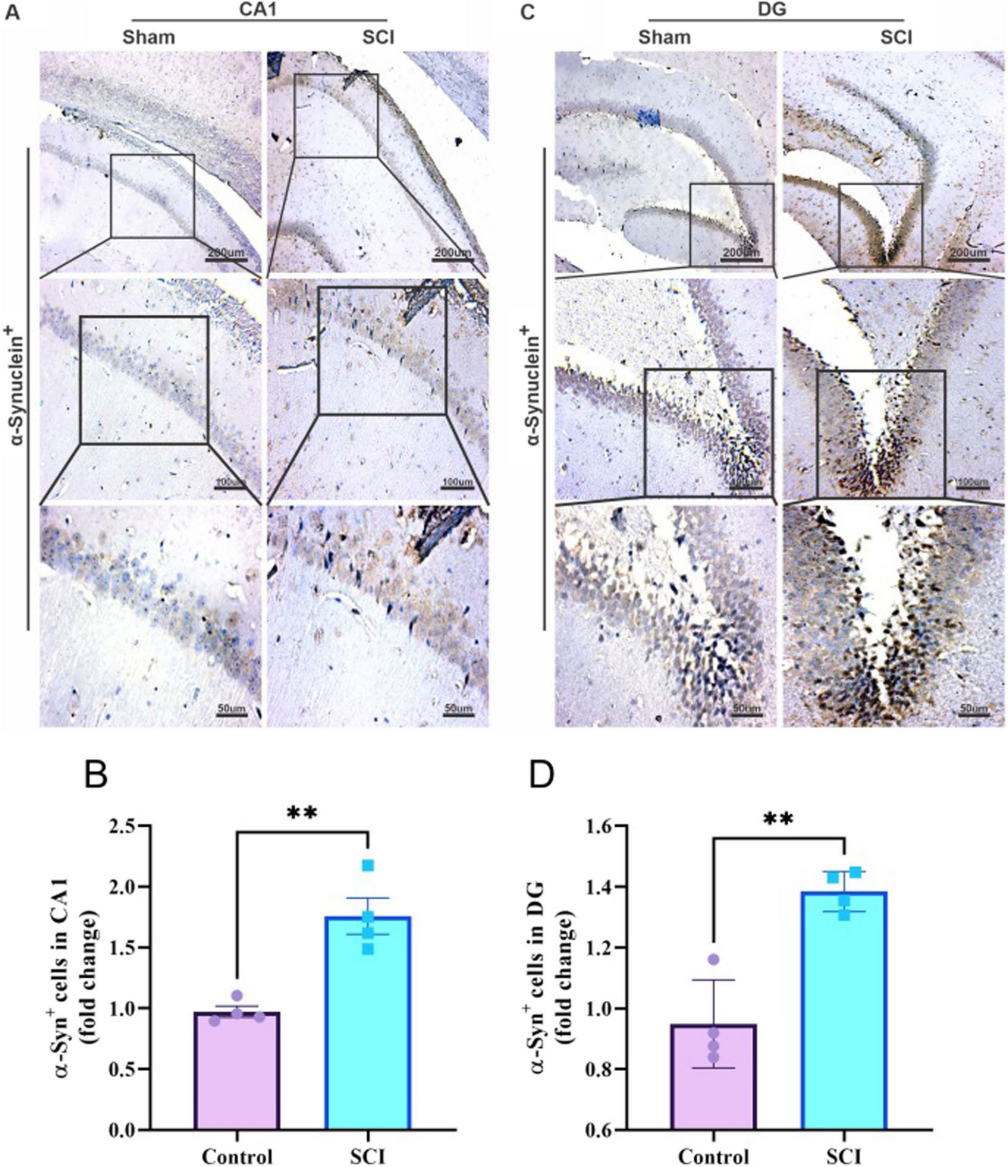
SCI increases the aggregation of α-synuclein in the CA1 and DG areas of the hippocampus. **A** Representative immunohistochemical images of alpha-synuclein aggregation in the CA1 region of the hippocampus. **B** Bar diagram shows that SCI significantly increase the accumulation of alpha-synuclein in the CA1 (n = 4 per group, **P < 0.01). **C** Representative immunohistochemical images of alpha-synuclein aggregation in the DG region of the hippocampus. **D** Bar diagram shows that SCI significantly increased the accumulation of alpha-synuclein in the DG (n = 4 per group, **P < 0.01)

As well as, the aggregation of α-synuclein (Fig. 7A and C) was significantly higher in the CA1 (P = 0.0024, t (6) = 5.037) (Fig. 7B) and DG (P = 0.0016, t (6) = 5.472) (Fig. 7D) areas of injured animals.

### SCI increases apoptotic cells in the hippocampus

**Fig. 8:**
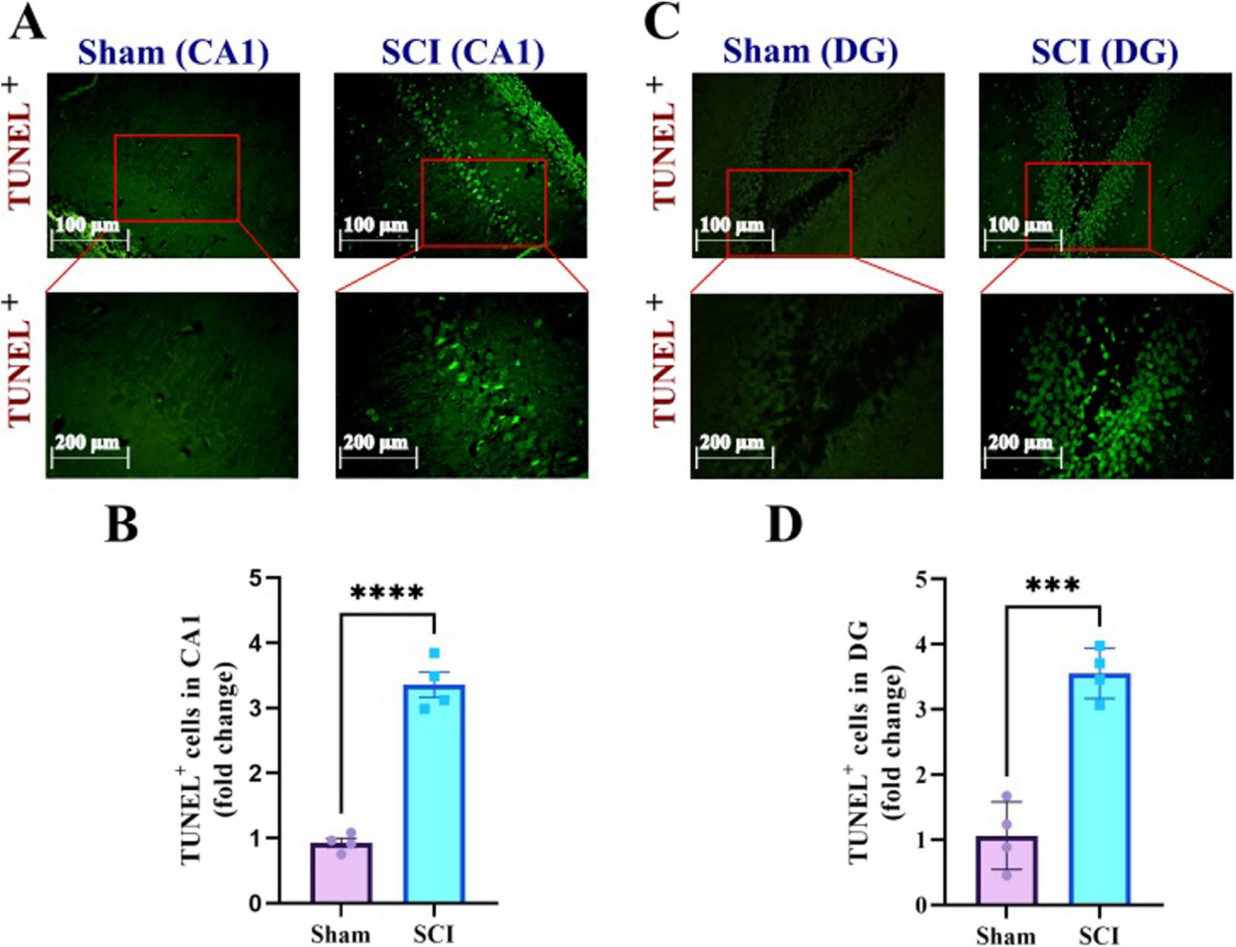
SCI increases the number of apoptotic cells in the CA1 and DG areas of the hippocampus. **A** Representative immunofluorescence images of TUNEL^+^ cells in the CA1 area of the hippocampus. **B** Bar diagram shows that SCI significantly increased the number of TUNEL^+^ cells in the CA1 (n = 4 per group, ****P < 0.0001). **C** Representative immunofluorescence images of TUNEL^+^ cells in the DG area of the hippocampus. **D** Bar diagram shows that SCI significantly increased the number of TUNEL^+^ cells in the DG (n = 4 per group, ***P < 0.001)

The rate of apoptotic cells in the CA1 and DG of the hippocampus was assessed using the TUNEL assay. Figure 8A and C, represents TUNEL^+^ in the CA1 and DG of sham and SCI groups. According to the independent t-test, there was a significantly higher apoptotic cells in the CA1 (P < 0.0001, t (6) = 11.79) (Fig. 8B) and DG (P = 0.0002, t (6) = 7.713) (Fig. 8D) of injured animals comparing with sham animals.

## Discussion

In the current study, approximately all the animals displayed cognitive deficits following SCI. This finding is in line with both human studies and experimental studies aiming to assess cognitive impairments following SCI in rodent models [23–26]. In the current study, with a new perspective, we sought to find the post-injury effects of SCI on the cognitive function of injured animals, parallel with alterations in the dopaminergic system, α-synuclein accumulation, neurogenesis, and apoptosis of the hippocampus. Using contusion model of SCI (mild injury), we demonstrated that after a mild injury of spinal cord, during the chronic phase of injury, there is an increase in the hippocampal apoptosis, deficiency in the hippocampal neurogenesis and expression of D1 and D5, as well as significant aggregation of α-synuclein accumulation in the hippocampal regions, on top of the gait and locomotion dysfunction. These defective manifestations of the hippocampus coincided with the dysfunction of the injured animals in the NORT and O-X maze test, as cognitive tasks. Considering the fact that locomotion ability is a basic necessity for cognitive tests, in this study, a mild contusion injury was utilized to minimize the effect of locomotion deficiency on the cognitive tests results.

One of the influential factors in the decline of cognitive function in various diseases of the nervous system is inflammation, which is associated with the accumulation of Tau or alpha-synuclein in different areas of the brain [27–29]. After the SCI, there is a progressive increase of tau pathology in the brain areas of rodents, parallel with the disruption of microtubule and mitochondrial structure, which lead to spatial memory deficits based on Y-maze spontaneous alteration test, and anxiety/risk-taking behavior deficits based on Elevated Plus Maze (EPM) test [30].

In a study by Brakel and colleagues, after the induction of SCI in rodents, the animals experienced depressive-like behaviors and expressed a significantly greater level of pro-inflammatory cytokines, accompanying a lower level of hippocampal neurogenesis compared to not-depressed subjects. Interestingly, the animals that later showed depressive manifestations had higher levels of IL-6, which continued throughout the experiment [31]. Secreted amyloid precursor protein alpha (sAPPα) from neurons triggers the release of IL-1 by activating primary microglia. On the other hand, there is a direct correlation between the elevated levels of α-synuclein and increased level of IL-1 [32]. In addition, incubation of microglia under the synuclein enriched conditions induces the over expression of COX2, NOX2, iNOS and TNF-α [33]. In the present study, the amount of alpha-synuclein in the animals with SCI was significantly higher than in the animals in the control group, which indicated the synergistic effect of possible inflammation of the hippocampus with the accumulation of alpha-synuclein in neural dynamics of the hippocampus.

Furthermore, α-synuclein aggregation impairs long-term potentiation [34, 35]. Findings have shown that after extended exposure of the hippocampus to the α-synuclein oligomers, they bind to the active site of NMDA receptors, and activate the calcium-permeable AMPA receptors, which in turn leads to resistance against the applied physiological stimuli. On this account, long-term potentiation (LTP) is impaired, which is one of the basic functions of the hippocampus in the course of learning and memory [36]. Confirming previous findings, the present study showed that alpha-synuclein accumulation has a destructive effect on animals’ memory and learning based on the indices of O-X maze and NORT tests, such that the injured animals spent more latency time finding the gifts in the O-X maze, and spent less time exploring the new object in the NORT.

In addition to alpha-synuclein accumulation, elevated hippocampal cell loss and apoptosis can affect cognitive function of animals [37]. Magnetic Resonance Imaging (MRI) studies have shown that, after the SCI, reduction of grey mater volume is not limited to the sensory-motor cortex, and grey matter volume of the hippocampus, parahippocampal gyrus, orbitofrontal cortex, pre-frontal cortex and anterior cingulate cortex progressively reduce [38–42]. Considering the close interaction between the hippocampus and other cognitive parts of the brain specially the prefrontal cortex [43], the synchronous communication of these parts can be affected after the SCI that have not been investigated in the present study.

According to previous studies, after the occurrence of SCI, along with the cell loss and inflammation different areas of the hippocampus, like CA1 and dentate gyrus, the spatial working memory impairs in rodents [25]. Herein, following the induction of SCI, parallel with the accumulations of alpha-synucleins in different areas of the hippocampus, the rate of cell death and apoptosis increased in those areas, which in turn could lead to a decline in cognitive function and neurogenesis of the hippocampus.

The occurrence of cell death in different parts of the hippocampus can be parallel with a remarkable increase in inflammatory cytokines (TNF-α, IL-6 and IL-1β) [44], and reduction in hippocampal neurogenesis after the SCI [45]. Through determination of the fate of hippocampal neural stem cells, various factors such as ASCL1, SOX2, DCX and, NeuN are expressed in different steps of matuartion [17, 46–48], whose expression may be affected due to SCI.

Induction of SCI in rodent models, triggers the hippocampal neurodegeneration by decreasing the proliferation and differentiation of neural stem cells to mature granular cells in the hippocampus [21]. Following SCI, the expression of DCX^+^ and NeuN^+^ cells decrease in the dentate gyrus coinciding with cell loss in the thalamus, cortex, and hippocampus and also elevated expression of inflammatory factors like translocator protein and chemokine ligand 21 (CCL21) (C–C motif), that consequently impairs the recognition of novel object in the NORT, and spatial memory in the MWM [49]. Decreased hippocampal neurogenesis and endoplasmic reticulum stress after the SCI, can lead to depressive-like behaviors, spatial memory deficets through the Y-Maze performance, and also unability to recognize novel objects through NORT performance [50]. In agreement with these findings, we indicated that after SCI the expression of DCX and SOX2, as a marker of stem cells and neurogenesis, declines significantly in the hippocampus simultaneously with impaired function of the animals in O-X maze and NORT. Ofcourse, considering the preseance of neuropathic pain and depression after the SCI, one of the interfering factors that may lead to the cognitive deficiency, is the possible neuropathic pain owing to injured spinal cord [2, 31, 51].

There is a harmonic crosstalk among multiple receptors homing in the hippocampus. This harmonious manifestation of receptors, is important through cognitive procesesse of the hippocampus, and also tunes the fate of hippocamal neural stem cells in the neurogenic region of the hippocampus, dentate gyrus [52–54]. NMDA receptors, has a facilitating function in most hippocampal neural circuits and regulates the proliferation of neural progenitor cells and differentiation of them to the adult ones [55]. Beside the hippocampal neurogenesis regulator, it has a pivotal role in the generation of rhythms like gamma and theta rhythms which are essesntial in the processs of dynamic information through cognitive tasks [56]. Morever, Muscarinic1 (M1) receptors have a crucial role in the neurogenci niche of the hippocampus, and following injection of the galantamine as an inhibitor of acetylcholinesterase, the proliferation and differentiation of stem cells improves in the DG [57]. The GABA A receptor, as an inhibitory receptor, is of great importance in modulating mutual interactions of the hippocampal regions [58]. GABAergic interneurons within the hippocampal microcircuits, inhibit the adjacent pyramidal neurons, and the release of GABA A from local interneurons preserves the quiescence state of stem cells, and regulates the differentiation of them to mature granule cells of the DG [59].

According to a recent study, following SCI, besides the decrease of receptors (NMDA, M1, and GABA A) expression in the hippocampus, there is an elimination of hippocampal neurogenesis and decrease of hippocampal theta power through spatial working memory task that proved by deficiency in the Y-maze performance [60]. In a similar studty, low expression of mentioned receptors after the SCI, decrease hippocampal neurogenesis and infulence the power and max-frequency of various hippocampal rhythms such as theta, delta, and gamma rhythms power, that are crucial in the process and neural dynamics of spatial memory [21, 61].

Nevertheless, along with the above mentioned receptors, the essential role of the dopaminergic receptors cannot be neglected, in the hippocampal circuits and cognitive functions. D1-like receptors of Dopamine (D1/D5) have been proven to be vital in the intercedeing of information salience and enhancing the persistence of long-term plasticity in hippocampus [62]. Activation of D1-like receptors by pharmacological agonism, enhances neural stem cells proliferation, deferentiation and long term survival through positively regulating of Wnt / β-catenin pathways, and it also applies anti-depressant and anti-anxiety effects [63].

Dopaminergic fibers of midbrain, sparsely innervate hippocamopal regions like DG, a receiver of convergent cortical inputs [64]. These inputs influence hippocampal dopamine receptors and can affect hippocampal cognitive function and neurogenesis [65, 66]. The activity of hippocampal D1 like receptors, mediates novelty-detection through the NORT, such that, post-training infusion of D1-like receptor antagonist not only significantly weakenes the tendency to explore novel objects, but also decreases the AMPA/NMDA ratio, stater of potentiated synaptic current [67]. The findings of the current study confirmed those reported by previous works, suggesting that the expression of D1-like receptors was significantly reduced after SCI, which may be involved in reduced neurogenesis and cognitive function. However, in addition to NMDA, M1, GABA A and dopaminergic receptors, that studied in the current study, the serotonergic system play a vital role in the cognitive function, synaptic plasticity, and hippocampal neurogenesis [68, 69], which can be examined in future studies.

In summary, it could be argued that following SCI, given the rise in hippocampal cell death, neurogenesis deficiency, alpha-synuclein aggregation, and dopmineric system deficiency, cognitive function of injured animals decline.

## Conclusions

**Fig. 9:**
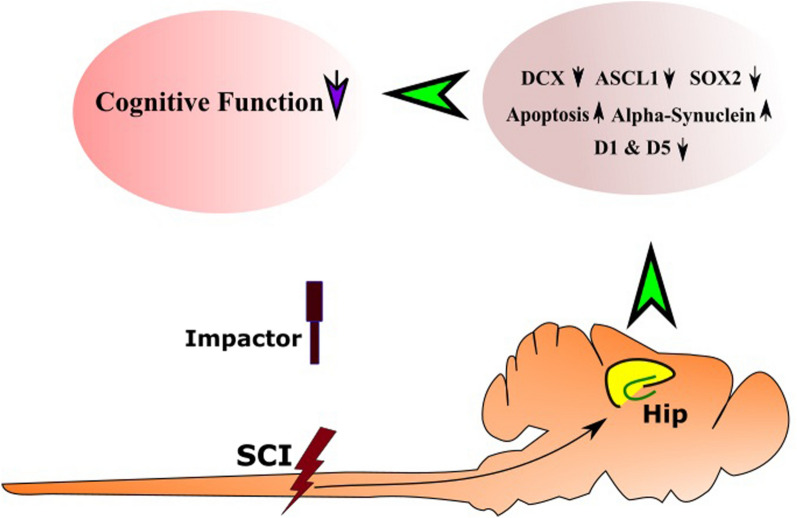
Graphical abstract

The present study offers an insight to the degenerative complication of SCI in the hippocampus (Fig. 9), so that this condition aside from apoptotic and inflammatory side effects in the hippocampus, thriggers the aggregation of alpha-synuclein in the hippocampal regions, and disorganize the dopaminergic system of the hippocampus. These disturbances go along the neurogenesis deficiency, and eventually manifests itself as a disorder in various cognitive functions such as learning, memory and recognition potency.

## Materials and methods

**Fig. 10:**
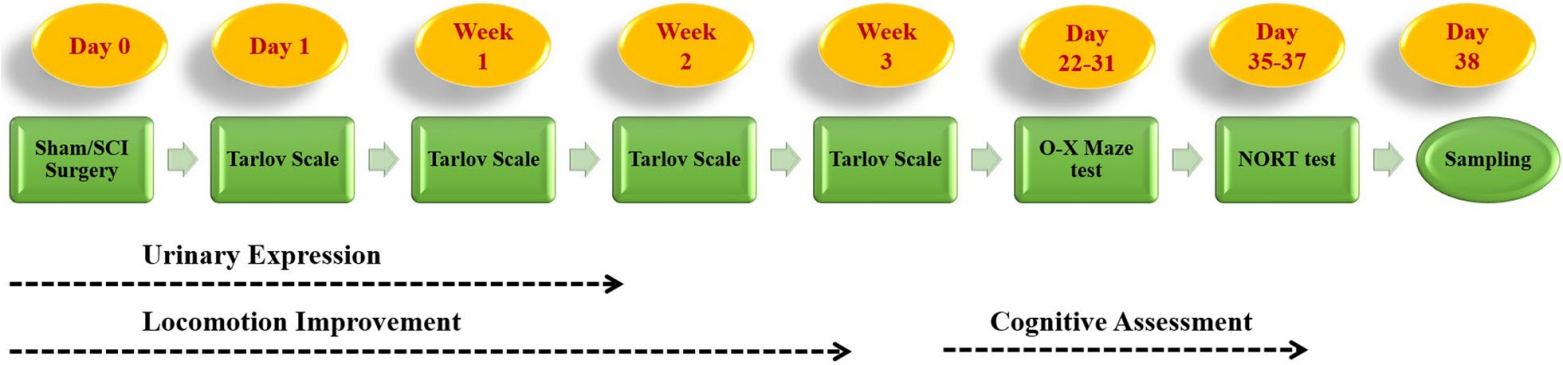
Experimental design

The experimental design of the study is presented briefly in the Fig. 10

### Ethics approval

All experimental and surgical procedures of this study were confirmed by the Ethics Committee of Tabriz University of Medical Sciences (IR.TBZMED.VCR.REC.1398.267).

### Animals

Sixteen adult male Wistar rats (250–280), were randomly divided into two groups: 1. Sham (n = 8, laminectomy at T10 without SCI), 2. SCI (n = 8, laminectomy at T10 with SCI). All animals were kept under a controlled environment (over a 12 h/12 h light/dark cycle), with easy access to water and food.

### Induction of SCI

Animals were anesthetized with Ketamine and xylazine (100 mg/kg, 5 mg/kg), and after the laminectomy of T10 vertebra, an acute mild injury (50 kdyn force) was applied to the spinal cord using Neuroscience Research Center (NSRC) Impactor [70]. The reason for using mild injury is that the animals have minimal damage, which enabled them to cooperate in cognitive assessments. Sham group underwent a laminectomy at T10 level. After the surgical procedure, injured animals’ bladder was expressed manually, for 7 consecutive days to prevent urinary retention and neurogenic bladder formation. For pain management, ketoprofen (5 mg/kg) was injected subcutaneously, and to inhibit bladder infection and post-surgical dehydration, Ciprofloxacin (9 mg/kg) was diluted in 2 ml saline and injected intraperitoneally, for five consecutive days. After three weeks and improving the locomotion of the injured animals, they were assessed and compared with intact animals, in terms of cognitive function.

### Locomotion assessment

The locomotion of the animals was assessed using the Basso, Beattie, and Bresnahan (BBB) scale and open field test.

#### BBB scale

The hind limb locomotion of the animals was assessed using the BBB scale, during three weeks after the induction of SCI. The BBB, is a 0–21 point scale, and as the animal locomotion improves, the point earned by the animal will also increase. In brief, animals were monitored in the open field for 4 min, and their locomotion was evaluated by a trained researcher. Before the induction of SCI, normal locomotion of rats was evaluated.

#### Open field

The locomotor activity of all rats was measured by open field test at the end of third week and prior to cognitive assessments. Animals were individually placed in the chamber (40 cm × 80 cm) to explore it freely for 10 min. The traveled distance and speed of locomotion was assessed with ToxTrac, a robust software for tracking animals [71].

### Cognitive assessments

At the end of the third week, with the improvement of motor function of the injured animals and with the minimization of their movement disorder, their cognitive assessments were performed using O-X Maze and Novel Object Recognition (NORT) tests.

#### O-X maze test

O-X Maze is a task for assessment of memory and learning [72]. It consists of a black box with dimensions of 30 cm × 60 cm × 60 cm. The floor of box is divided by grid lines into 25 squares (12 cm × 12 cm). Four black blocks (10 cm × 10 cm × 10 cm) are placed in squares. In each block, holes are made in the center of four sides with a diameter and depth of 2 cm × 2 cm, and each hole is marked by one of the four symbols: O, X, =, II (Fig. 2A). Two days before the start of test, the rats received popcorn as a desired reward for taste habituation. Twenty-four hours before the start of the test, rats were subjected to dietary restriction (90% reduction from normal), and habituated to the box for 10 min. The total task period is 10 days and the number of tests for each rat is once a day and each time is ten minutes. The reward is placed inside the hole in position “O”. Rat is placed in the center of maze and given ten minutes to explore and find the reward. For all 10 days the reward is in the “O” position and the location of blocks changes every day (clockwise). The box, and blocks were cleaned with 70% alcohol through trials intervals to prevent confounding effects of odor. In this test, the following parameters were examined: (1) Time to find the first reward (first gift latency); and (2) Test completion time or total latency (finding all four rewards). For all animals, the maximum time will be 10 min. It should be noted that in some evaluation sessions, especially first session, the animals did not succeed in completing the test within 10 min, so we rated the total latency score according to Table 4.

**Table 4:** The O-X maze total latency scores

Function	Score
Unable to complete the task within 10 min	0
Completion of task within the first minute	10
Completion of task within the second minute	9
Completion of task within the third minute	8
Completion of task within the fourth minute	7
Completion of task within the fifth minute	6
Completion of task within the sixth minute	5
Completion of task within the seventh minute	4
Completion of task within the eighth minute	3
Completion of task within the ninth minute	2
Completion of task within the tenth minute	1

#### Novel object recognition (NORT) test

The NORT (Fig. 4A) [73] was conducted after the O-X maze test. Briefly, rats were habituated to the Open Field for 5 min, at the first day. Next day, two similar objects are placed in the right and left corners of the open field and the animal is allowed to explore freely in the apparatus for 5 min (sample phase). On the third day, one of the two similar objects is replaced with a novel object that is morphologically different from the previous object, to evaluate the object recognition in animals (choice phase). At this phase, the amount of time that each animal is exploring objects is recorded until the animal's ability to recognize is assessed. After testing each animal, the open field was cleaned with 70% alcohol to prevent confounding effects of animals’ odor. Based on the explorative inherit of animals, it’s expected that they spend more time to explore the new object that reflects the animal's intact memory.

### Western blotting

Western blotting was conducted pursuant to our previous studies [21]. Briefly, to assess the expression of D1 receptor, D2 receptor, ASCL1 and SOX2, the hippocampal tissues were homogenized by radioimmunoprecipitation assay (RIPA) buffer containing a protease inhibitor cocktail. The protein content of samples was separated using sodium dodecyl sulfate–polyacrylamide (SDS–PAGE, 12.5%) and transferred on to the polyvinylidene difluoride (PVDF) membranes. Next, membranes were blocked with 5% skim milk for 2 h at room temperature in Tris-buffered saline (TBS). The membranes were incubated at 4 °C overnight with primary monoclonal antibodies, anti D1 receptor, D5 receptor (sc-65314), SOX2 (sc-365823), and ASCL1 (ab107046).

The membrane was washed with TBS and next incubated for 1 h with horseradish peroxidase-conjugated (HRP)-labeled secondary antibodies. Eventually, the protein band intensities were detected using Image J software, and all bands normalized upon the β-actin band.

### Immunohistochemistry

The rats were sacrificed, and brains were removed from skull and fixed in 10% formalin, over nightly. Then formalin-fixed brain tissues embedded in paraffin and sectioned coronally (5 μm sections). We selected the comparable coronal sections displaying the same cytoarchitectonic characteristics, determined using the Paxinos and Watson atlas (The Rat Brain in Stereotaxic Coordinates—The New Coronal Set by George Paxinos, Charles Watson, Academic Press; 5th edition, November 10, 2004), in the injured (n = 4) and control (n = 4) rats. Every fourth section (3 sampled sections) was selected and analyzed for DCX^+^ and α-synuclein^+^ cells in the CA1 and DG regions.

Immunohistochemistry staining was conducted through the streptavidin–biotin method using antibodies against α-synuclein (sc-53955), and DCX (sc-8066). The sections were deparaffinized by xylene and then dehydrated in ethanol. Microwave irradiation was used to retrieve antigen. Slides were cooled at room temperature, then brain sections were incubated (4 °C, overnight) with primary antibodies of DCX and α-synuclein. After three time washing with TBS, sections were incubated for 1 h at room temperature with the secondary biotinylated antibodies and peroxidase-conjugated streptavidin. Finally, after the washing of sections, counterstaining was done with hematoxylin. The optical density of proteins in the CA1 and DG regions was determined by image j software, so that after the color segmentation and making binary, the intensity of color is measured by the software.

### Tunel assay

As in our previous studies [74], apoptotic changes in the hippocampus of injured (n = 4) and control (n = 4) animals were assessed by proteinase k and using tunel (terminal transferase-mediated dUTP nick end-labeling) staining (Sigma-Aldrich, Germany). Briefly, paraffin embedded sections (3 sampled sections like immunohistochemistry) were incubated with proteinase K (20 μg/ml), and after being covered with a buffer, washed three times. Finally, slides incubated (1 h at 37 °C) in the tunel staining compound (Terminal Deoxynucleotidyl Transferase (TdT) and fluorescein-12-dUTP). Shiny green cells (apoptotic ones) were observed in the CA1 and DG regions, using a fluorescence microscope (Zeiss AxioImager, Germany) and analyzed by Image-J software.

### Statistical analysis

All statistical analyses were done using the GraphPad Prism (Version. 9.0). Immunohistochemical data (DCX+ cells), Tunel+ cells, and relative density of proteins (D1, D5, ASCL1, and SOX2) that determined by western blotting, were analyzed using the independent t-test. The BBB scale scores, and first gift latency/total latency scores of O-X maze were analyzed by two-way ANOVA analysis, also, the multi-comparison analysis was used to analyze the intra-group difference. The similar object latency, novel object latency and, discrimination index of NORT were analyzed by independent t-test. The linear regression and Pearson correlation was used to determine whether hind limb locomotion (BBB scores) of SCI rats, predict the function of them in cognitive tasks. P < 0.05 was statistically significant. Using IBM SPSS.24 software, BBB score was used as a covariate in the ANCOVA, to determine whether it adjusts cognitive function of animals in the O-X maze and NORT.

## Data Availability

All statistical results are stated in the article, and raw data are available from the corresponding author on reasonable request.

## Abbreviations

ASCL1: Scute family BHLH transcription factor 1
BBB: Basso, Beattie, and Bresnahan
CA1: Cornu ammonis1
CCL21: Chemokine ligand 21
CDK: Cyclin dependent kinase
DCX: Doublecortin
DG: Dentate gyrus
D_1_ and D_5_: Dopaminergic receptor 1 and dopaminergic receptor 5
EPM: Elevated plus maze
HRP: Horseradish peroxidase
IL: Interleukin
M1: Muscarinic1
MWM: Morris water maze
NORT: Novel object recognition test
NSRC: Neuroscience Research Center
PVDF: Polyvinylidene difluoride
sAPPα: Secreted amyloid precursor protein alpha
SCI: Spinal cord injury
SGZ: Sub-granular zone
SOX2: SRY (sex determining region Y)-box 2
TBS: Tris-buffered saline
TNF-α: Tumor necrosis factor-α
Tunel: Terminal transferase-mediated dUTP nick end-labeling
